# Treatment for Intramuscular Lipoma Frequently Confused with Sarcoma: A 6-Year Restrospective Study and Literature Review

**DOI:** 10.1155/2014/867689

**Published:** 2014-12-10

**Authors:** Hyun Ho Han, Jong Yun Choi, Bommie F. Seo, Suk-Ho Moon, Deuk Young Oh, Sang Tae Ahn, Jong Won Rhie

**Affiliations:** Department of Plastic and Reconstructive Surgery, Seoul St. Mary's Hospital, College of Medicine, The Catholic University of Korea, 222 Banpo-Daero, Seocho-gu, Seoul 137-701, Republic of Korea

## Abstract

*Introduction.* Intramuscular lipoma is a very rare form of lipoma, known to be categorized as an infiltrating lipoma due to its tendencies to infiltrate the muscle or the synovium. Contrary to other subcutaneous lipomas, even after surgical removal, the rate of local recurrence ranges at a high rate from 50∼80% and differential diagnosis with liposarcoma is very difficult. *Patients and Methods.* A retrospective chart review was conducted for a total of 27 patients. Before performing a surgery based on the types of mass, a radiologic imaging study was performed. An intraoperative frozen biopsy was performed on every patient and the results were compared. The progress was monitored every 3 to 6 months for recurrence or struggles with rehabilitation. *Results.* There were 13 male and 14 female patients with an average age of 54.6. The average tumor size was 8.2 cm (1.1 cm∼31.6 cm). Excision was performed using a wide excision. All 27 individuals were initially diagnosed as intramuscular lipoma; however, 1 of the patients was rediagnosed as liposarcoma in the final checkup. The patients had an average of 3 years and 1 month of follow-up and did not suffer recurrences. *Conclusion.* Thus, it is essential that a frozen biopsy is performed during the surgery in order to identify its malignancy. And a wide excision like malignant tumor operation is a principle of treatment.

## 1. Introduction

Intramuscular lipoma is a very rare form of lipoma, located deep within the muscle fibers and showing characteristics of infiltrating the muscles surrounding the area. Although it is not clear about the origin of tumors, a wide variety of theories, such as metaplasia, trauma, chronic irritation, and congenital development, have been suggested [1–3]. There have not been many instances of intramuscular lipoma reports due to its rare occurrence rates. Even in those cases, they were of specific muscle locations and were more of a simple case report. Further, there has not been many journals consisting of a wide variety of clinical data and not shown any treatment guideline. In order to upgrade a protocol to treating intramuscular lipoma, we have compared the results from our center to other centers and conducted a retrospective chart review for a total of 27 patients from January 1, 2007, to January 1, 2013.

## 2. Materials and Methods

A chart review was performed on a total of 27 patients from January 1, 2007, to January 1, 2013. Each patient had a mass ranging all over the body and had visited a local clinic prior to transferring to our facility. Three of 27 patients had experienced an excision surgery at a local clinic and had relapsed. For all patients, a wide range excision surgery and a biopsy were performed.

### 2.1. Preoperative Evaluation

Prior to the surgery, a CT or MRI was conducted in order to determine the size and location of the mass. The patients were also asked about any trauma relating to mass, history, and other symptoms.

### 2.2. Operation Procedure

Considering the characteristics of intramuscular lipoma, in order to stop the possibility of recurrence, a wide excision was performed just as if it were for a malignant tumor. An excision was performed 1 cm from the lipoma margin, starting from the healthy muscle margin. In order to reduce the operation site morbidity, the remaining muscle fibers were repaired strictly as it could be based on approximations. In all cases, an intraoperative frozen biopsy was performed afterwards in order to determine the malignancy.

### 2.3. Frozen Biopsy

After a total excision is performed, the whole specimen is sent to the pathologist. Pathologist makes a gross examination of the specimen and checks mainly for portions that are suspected of malignancy, as well as tumor margins. If the specimen margin finds a malignancy cell or suspects of one, a minimum of 1 cm safety margin is acquired by performing further excision.

### 2.4. Postoperative Management and Follow-Up

After surgery, most of the patients had no complications and were discharged from the hospital. One male patient who had a final diagnosis of liposarcoma was referred to the oncology department and had 3 months of radiation therapy performed (2012.10.31~2012.12.11, total 6000 Gy). The patient was monitored for 3–6 months for any recurrence; in addition, the area surrounding the excision was monitored and checked for a range of motions.

### 2.5. Typical Clinical Case

**Figure 1:**
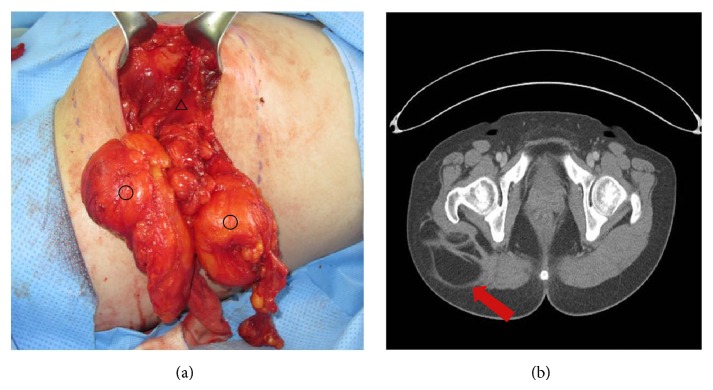
Case 12. (a) Intraoperative clinical photography. (○: intramuscular lipoma, △: gluteus maximus muscle). (b) CT image shows a 6.5 × 11.0 cm sized infiltrating lipomatous mass in the Rt. gluteus maximus muscle.

The patient (case 12) was a 32-year-old female, who was admitted to the center due to a tumor that was increasing throughout a 2-year period, located on the right buttock. The patient had no special complaints other than asymmetric buttocks. The tumor was soft when touched and was sized 11 × 6.5 cm; moreover, it did not have clear boundaries (Figure 1).

A frozen biopsy was performed during the surgery in order to rule out the possibility of sarcoma and a nonmalignant result was established. After surgery, a patient was diagnosed as having intramuscular lipoma and was completely healed with no wound problems. She was checked throughout about 3-year period but did not suffer a recurrence.

## 3. Results

**Figure 2:**
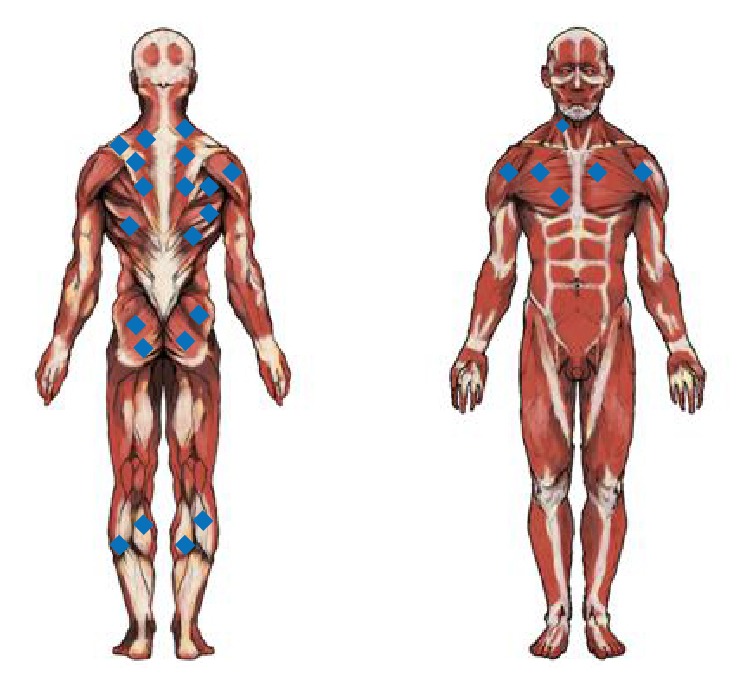
Sites of intramuscular lipoma. Distribution of intramuscular lipoma depending on the muscle where it occurred. It occurred mostly in muscles, such as the deltoid muscle, latissimus dorsi muscle, trapezius muscle, pectoralis muscle, and gluteus maximus muscle, which are large muscle groups. Four cases (15.4%) in the lower body and 22 cases in the trunk and upper body (84.6%).

Patient data is summarized in Table 1. There were a total of 27 patients, (13 male, 14 female) with an average age of 54.6 (age 30 to 72). 18.5% (5/27) complained of pain in the muscle, while the remaining 22 patients, or 81.5% (22/27), had no other symptoms other than the tumor being felt. The muscle where intramuscular lipoma existed follows (Figure 2). The deltoid muscle, latissimus dorsi muscle, trapezius muscle, pectoralis muscle, gluteus maximus muscle, gastrocnemius muscle, and other large muscle groups within the body are where the tumor commonly occurred (Table 2). The common tumor size was 8.2 cm (1.1 cm~31.6 cm). In terms of the ratio of where this occurred, 4 cases were in the lower extremities and 22 cases were in the trunk and upper body (84.6%). In every case, prior to surgery, a CT or MRI was scanned; however, based on the radiology tests, a diagnosis of lipoma or liposarcoma could not be confirmed.

**Figure 3:**
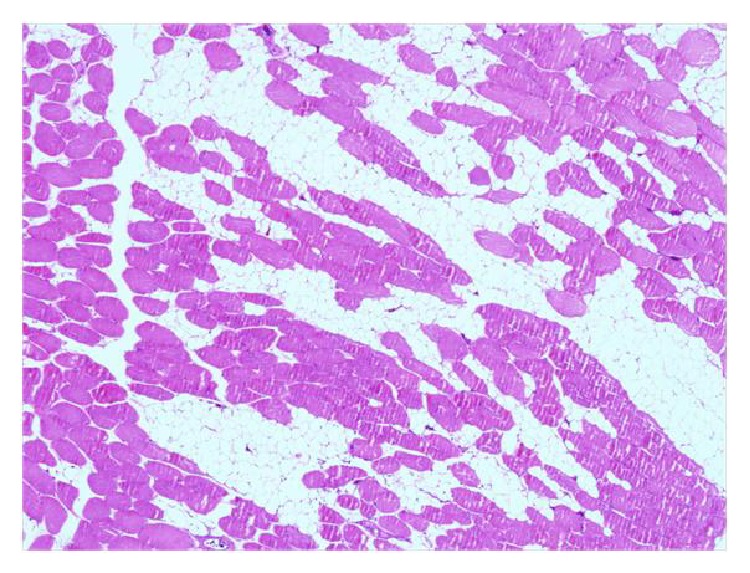
Histopathology for intramuscular lipoma. (H&E stain ×100). Fat cells are infiltrating the muscles in the form of stria. The tumor showed mature adipocytes, no lipoblasts, and normal or slightly atrophic muscle fibers. Mature univacuolated lipocytes of a fairly uniform size are seen.

**Figure 4:**
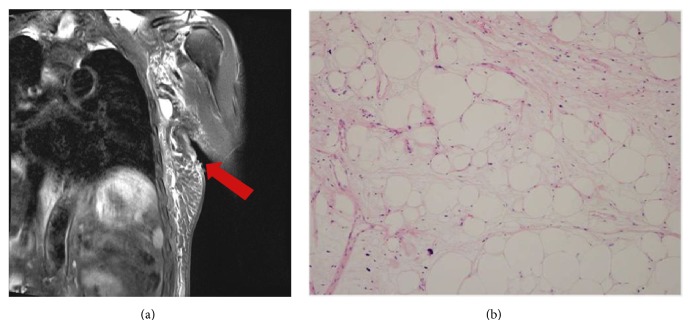
Case 27. (a) MRI shows well-differentiated mass infiltrating the pectoralis major muscle and lattisimus dorsi muscle. (b) Histopathology for liposarcoma (H&E stain ×100) multivacuolated lipoblasts, cellular pleomorphism, marked vascularization, and mitotic activity, which are the differences with intramuscular lipoma.

In the biopsy results, during surgery, the frozen biopsy showed a nonmalignant result for all 27 participants; however, one patient showed a liposarcoma in the permanent results with a free tumor margin. Only 1 patient out of 27 patients, or 3.7% (1/27), who were suspected of intramuscular lipoma was diagnosed as liposarcoma, whereas the remaining 26 patients, or 96.3% (26/27), were finally diagnosed as intramuscular lipoma. During surgery, unlike other subcutaneous lipomas, there were no capsules typically surrounding the tumor. What was found was that, within the muscle fibers, lipomatous tissues were infiltrating within the muscle fibers (Figure 3). Prior to surgery, the patient finally diagnosed as liposarcoma showed an infiltrating tumor like other intramuscular lipomas within the pectoralis major muscle and lattisimus dorsi muscle when an MRI was performed; however, malignancy could not be identified, which was later diagnosed as liposarcoma by the permanent final results (Figure 4).

For all patients, the recurrence rate was taken into consideration and a follow-up was conducted every 3 to 6 months. Patients had an average follow-up duration of 3 years and 1 month (1 year–6 years); in this period, no recurrence was found (recurrence rate of 0%). However, considering that 3 patients had experienced recurrence after visiting their local clinics, the recurrence rate could be considered to be 11% (3/27). More than half the patients, or 15 of the 27, complained of muscle aches in the region of surgery; yet, they did not experience any problems within the joints or daily routines and they recovered completely.

## 4. Discussion

Intramuscular lipoma is a very rare form of lipoma occurring in approximately 1% of all lipomas [4]. Recurrence rates differ based on the reports but typically range from 50 to 80% [5]. This is due to its condition where it infiltrates within the muscle fiber; thus, a total excision is made difficult. Within the center of this report, the recurrence rate was 0%; if procedures including other facilities are included, the recurrence rate was 11.1% (3 of 26). When the principle of wide range excision was kept when performing the excision, contrary to the known data, we were able to reduce the recurrence rate to 0%.

Because of infiltrating characteristics, patients may complain of pain in certain movements within the muscle. At our center, patients had complained of pain in 18.5% (5 of 27), which is a higher rate than in former reports, where 100% of the patients reported no symptoms [6]. There have been reports of a case where intramuscular lipoma formed in the supraspinatus muscle causes impingement syndrome [7].

According to Kindblom et al. [8] and Enzinger and Weiss [9], intramuscular lipoma generally appears in a large form bigger than 5 cm, located below the deep subfascia. Further, it is generally in large muscle groups, such as the shoulder, upper arm, hip, and thighs. Our findings in our center were consistent with this, where the average size was 8.2 cm and it was located in large muscle groups, such as trapezius muscle, deltoid muscle, latissimus dorsi muscle, pectoralis muscle, gluteus maximus muscle, and gastrocnemius muscle. In the upper extremity and the trunk, it occurred most commonly in the trapezius muscle, the deltoid muscle, and the pectoralis major muscle. In the buttocks and lower leg, it occurred most commonly in the gluteus maximus muscle and gastrocnemius muscles, which are generally large muscles. If we were to speculate as to why they are found in large size, it is because they are located deep within the muscles; thus, they are found later than most general lipomas. We also assumed that intramuscular lipoma occurred with a high probability of injuring muscles that are large groups where the joint's range of motion is big, and much power is used; this supports the idea that trauma like a muscle tear is a possible starting point for intramuscular lipoma [10, 11].

Generally when soft tissue tumor exists throughout the body in large sizes, if deeply located and infiltrating, a diagnosis of a soft tissue sarcoma must be considered [12]. At our center, 3.7% (1/27) of the patients with intramuscular lipoma were also diagnosed with liposarcoma. Until a histological diagnosis was confirmed, the surgery was planned under the assumption of liposarcoma. In one case, a mass was determined to be a benign lipoma after an intraoperative frozen biopsy; however, a final diagnosis after surgery proved to be liposarcoma and thus, until a final histological exam is made, malignancy cannot be ruled out. Hence, our center suggested a 1 cm safety margin while performing a wide excision. Of course, if vital organs or important vessels or nerves are nearby, marginal excision is performed inevitably and if malignancy is confirmed, radiotherapy must be further performed.

Currently, the principle of treating low grade and well-differentiated sarcoma is to perform a surgical total excision with a negative margin, and for intermediate to high grade and poorly differentiated sarcoma, additional safety margin of 1 cm is required. In the instance of a safety margin of 1 cm not being possible, radiotherapy or extra excision is required [13–15]. In case 27, where the patient was diagnosed as liposarcoma, we had performed an excision with a safety margin of 1 cm, however, after a histopathology analysis which revealed that a 1 cm safety margin was not acquired, thus following the protocol mentioned above. So radiotherapy was performed at adjuvant of a total of 6000 Gy. Chemotherapy in the treatment of liposarcoma is still not being proved [13].

Berquist et al. [16] claimed that the bigger the size of the tumor, the higher the possibility that the tumor is malignant. In order to support their claims, they demonstrated that, in their cases of 95 different benign and malignant soft tissue masses and in masses sized smaller than 3 cm, not one malignant tumor was found. Our case diagnosed with liposarcoma was sized 11.8 × 4 cm, which was much larger than our total average size of 8.4 cm, this point also supports the idea that a huge lipoma would have to consider the possibility of malignancy. However, the final determination always must be done through a histopathological examination [17]. As you can see in Figure 3, intramuscular lipoma is present in showing the well-differentiated adipose tissue as well as the penetrating normal or slightly atrophic muscle fiber; further, a mature adipocyte with identical sized univacuole was observed. Out of these, there is minimal nuclear atypia and if fewer or no lipoblast is observed, we can diagnose as an atypical lipoma [18].

On the contrary, liposarcoma, as shown in Figure 4, shows multivacuolated lipoblasts, cellular pleomorphism, marked vascularization, and mitotic activity. If a cytogenetic analysis can be performed, a giant ring and marker chromosomes composed of the q12-15 region of the chromosome are shown and thus, we can make a diagnosis of sarcoma [19].

There is some controversy around using a wide excision during surgery; however, the first reason as to why a wide excision principle must be done is because of the possibility of malignancy and, second, high recurrence rates. Recurrence rates varied based on reports: 80% [5] and 62.5% [6]; so other reports have also claimed the importance of a wide excision like us [6, 11]. However, others, such as Kawaguchi et al. [12], were against using excision with a wide margin just because of the mass being a benign soft tissue tumor and, instead, preferred intralesional curettage. However, after performing intralesional curettage and recurrence was found, the danger is that even more muscles will be cut off; thus, operation site morbidity could be higher. And if final diagnosis was confirmed to the liposarcoma, reoperation should be needed and adjuvant radiotherapy would be delayed.

At our center, before surgery, we performed an MRI (7.4%) or CT (92.6%) scan for every case. The opinion of the radiologist was that, in all cases, intramuscular lipoma looked apparent, but liposarcoma could not be completely ruled out. In the case of the patient who was diagnosed as liposarcoma in the histological test, lipoma was suspected in the initial MRI before the surgery. It is difficult to determine if it is malignant or benign through a CT or MRI scan. According to Nishida et al. [17], intramuscular lipoma is a (1) lesion located within the muscle and (2) a fat density lesion containing thick soft tissue density streaks of variable thickness and occasional interruption, representing the entrapped muscle fibers. These characteristics are consistent with opinions of radiologists of it being a soft tissue sarcoma. In the case of liposarcoma, there are instances of a CT scan showing a fair hazy amorphous area and soft tissue density septae, or T2 weighted image showing the fat to be high intensity relative to normal levels (shown as the same density in the T1 weighted image) which can help with diagnosis but there are limitations due to lack of consistency.

Although it is difficult to tell if it is malignant or benign through radiological tests, the size, depth and area of the mass can be just found through a CT or MRI scan. It is particularly useful to determine the conditions, such as haematoma, synovial cysts or muscle tears [20].

As aforementioned, once intramuscular lipoma is suspected, a wide ranged excision must be performed. First, the infiltrating tendencies within the muscle fiber make the recurrence rates to become high; second, prior to the surgery, a radiologic study image does not help differentiate soft tissue malignancy. Thus, a final biopsy must be conducted. Because intramuscular lipoma is very rare, it is difficult to gather massive amounts of data for review. Therefore, we are hoping that this report can act as a guide in treating intramuscular lipoma.

## 5. Conclusion

Intramuscular lipoma has infiltrating tendencies and high recurrence rates. Further, it is difficult to differentiate sarcoma prior to surgery. Radiological tests prior to surgery will not determine its malignancy. During surgery, a frozen biopsy must be performed in order to find out the malignancy; until the final biopsy results are out, malignancy cannot be ruled out. Authors used wide excision like a malignant tumor as a treatment principle and were able to dramatically reduce the recurrence rates.

## Figures and Tables

**Table 1:** Patients data.

Patient	Sex	Age	Size (cm)	Site of lipoma	Preoperative evaluation	Preoperative symptoms	Frozen biopsy	Follow-up	Recurrence	Limit of motion	Final diagnosis
Pt 1	M	65	9.3 × 4.4	Trapezius m.	CT	Pain and tenderness	Nonmalignancy	6 Y	None	None	Lipoma
Pt 2	M	66	11.5 × 7.3	Gastrocnemius m.	CT	Bulging mass	Nonmalignancy	4 Y	None	None	Lipoma
Pt 3	F	61	10.8 × 8.4	Trapezius m.	CT	Bulging mass	Nonmalignancy	3 Y 6 M	None	None	Lipoma
Pt 4	F	58	5.5 × 3.8	Gluteus maximus m.	CT	Bulging mass	Nonmalignancy	4 Y	None	None	Lipoma
Pt 5	M	45	17.0 × 6.5	Trapezius m.	CT	Bulging mass	Nonmalignancy	4 Y 3 M	None	None	Atypical lipoma
Pt 6	M	59	31.6 × 6.0	Latissimus dorsi m.	CT	Tenderness and bulging mass	Nonmalignancy	4 Y 2 M	None	None	Atypical lipoma
Pt 7	F	46	2.5 × 1.7	Pectoralis major m.	CT	None	Nonmalignancy	4 Y 4 M	None	None	Lipoma
Pt 8	F	51	6.0 × 5.0	Gastrocnemius m.	CT	Palpable mass	Nonmalignancy	3 Y 3 M	None	None	Lipoma
Pt 9	M	51	3.2 × 4.7	Trapezius m.	CT	Tenderness	Nonmalignancy	3 Y 2 M	None	None	Lipoma
Pt 10	M	57	6.7 × 4.0	Rhomboid major m.	MRI	Palpable mass	Nonmalignancy	3 Y	None	None	Lipoma
Pt 11	F	57	4.4 × 2.4	Latissimus dorsi m.	CT	None	Nonmalignancy	3 Y 2 M	None	None	Lipoma
Pt 12	F	32	6.5 × 11.0	Gluteus maximus m.	CT	Bulging mass	Nonmalignancy	3 Y 3 M	None	None	Lipoma
Pt 13	F	69	3.2 × 2.1	Gastrocnemius m.	CT	None	Nonmalignancy	4 Y 9 M	None	None	Lipoma
Pt 14	M	51	1.1 × 2.3	Pectoralis major m.	CT	None	Nonmalignancy	3 Y 8 M	None	None	Lipoma
Pt 15	M	48	7.2 × 3.4	Gastrocnemius m.	MRI	Bulging mass	Nonmalignancy	2 Y 6 M	None	None	Lipoma
Pt 16	F	59	7.2 × 3.0	Latissimus dorsi m.	CT	None	Nonmalignancy	3 Y 4 M	None	None	Lipoma
Pt 17	M	58	1.5 × 2.3	Trapezius m.	CT	Diffuse pain	Nonmalignancy	3 Y	None	None	Lipoma
Pt 18	F	75	3.5 × 1.5	Gluteus maximus m.	CT	None	Nonmalignancy	2 Y 2 M	None	None	Lipoma
Pt 19	F	47	8.7 × 2.6	Deltoid m.	CT	Bulging mass	Nonmalignancy	3 Y 3 M	None	None	Atypical lipoma
Pt 20	F	50	3.5 × 5.0	Rhomboid major m.	CT	Diffuse pain	Nonmalignancy	1 Y 6 M	None	None	Lipoma
Pt 21	F	64	7.8 × 2.2	Platysma m.	CT	Palpable mass	Nonmalignancy	2 Y 4 M	None	None	Lipoma
Pt 22	M	51	8.4 × 2.5	Deltoid m.	CT	Palpable mass	Nonmalignancy	2 Y 2 M	None	None	Lipoma
Pt 23	M	48	4.4 × 3.5	Gluteus maximus m.	CT	None	Nonmalignancy	1 Y	None	None	Lipoma
Pt 24	F	41	5.4 × 2.9	Trapezius m.	CT	Palpable mass	Nonmalignancy	1 Y 8 M	None	None	Lipoma
Pt 25	F	30	1.4 × 1.5	Pectoralis major m.	CT	None	Nonmalignancy	1 Y 6 M	None	None	Lipoma
Pt 26	M	64	1.5 × 7.2	Deltoid m.	CT	None	Nonmalignancy	2 Y	None	None	Lipoma
Pt 27	M	72	11.8 × 4.0	Latissimus dorsi m.	MRI	Bulging mass	Nonmalignancy	1 Y 6 M	None	None	Liposarcoma

**Table 2:** Sites of intramuscular lipoma.

Muscle	Number of lesions
Trapezius m.	6
Gastrocnemius m.	4
Gluteus maximus m.	4
Latissimus dorsi m.	4
Pectoralis major m.	3
Deltoid m.	3
Rhomboid major m.	2
Platysma m.	1

Total	27
